# Major Cardiovascular Risk Factors in Latin America: A Comparison with the United States. The Latin American Consortium of Studies in Obesity (LASO)

**DOI:** 10.1371/journal.pone.0054056

**Published:** 2013-01-17

**Authors:** J. Jaime Miranda, Victor M. Herrera, Julio A. Chirinos, Luis F. Gómez, Pablo Perel, Rafael Pichardo, Angel González, José R. Sánchez, Catterina Ferreccio, Ximena Aguilera, Eglé Silva, Myriam Oróstegui, Josefina Medina-Lezama, Cynthia M. Pérez, Erick Suárez, Ana P. Ortiz, Luis Rosero, Noberto Schapochnik, Zulma Ortiz, Daniel Ferrante, Juan P. Casas, Leonelo E. Bautista

**Affiliations:** 1 CRONICAS Center of Excellence in Chronic Diseases, Universidad Peruana Cayetano Heredia, Lima, Peru; 2 Department of Medicine, School of Medicine, Universidad Peruana Cayetano Heredia, Lima, Peru; 3 Department of Population Health Sciences, University of Wisconsin, Madison, Wisconsin, United States of America; 4 Universidad Autónoma de Bucaramanga, Bucaramanga, Colombia; 5 University of Pennsylvania and Philadelphia VAMC, Philadelphia, Pennsylvania, United States of America; 6 Pontificia Universidad Javeriana, Bogotá, Colombia; 7 Faculty of Epidemiology and Population Health, London School of Hygiene and Tropical Medicine, London, United Kingdom; 8 Instituto Dominicano de Cardiología, Santo Domingo, República Dominicana; 9 Centro Nacional de Alimentación y Nutrición, Instituto Nacional de Salud, Lima, Peru; 10 Departamento de Salud Pública, Pontificia Universidad Católica de Chile, Santiago, Chile; 11 Ministerio de Salud de Chile, Santiago, Chile; 12 Instituto de Investigación y Estudios de Enfermedades Cardiovasculares, Facultad de Medicina, Universidad del Zulia, Maracaibo, Venezuela; 13 Cardiovascular Diseases Epidemiologic Observatory, Epidemiologic Research Center, Universidad Industrial de Santander, Bucaramanga, Colombia; 14 Santa María Catholic University School of Medicine and Santa María Research Institute, Arequipa, Peru; 15 Department of Biostatistics and Epidemiology, Graduate School of Public Health, Medical Sciences Campus, University of Puerto Rico, San Juan, Puerto Rico; 16 Universidad de Costa Rica, Centro Centroamericano de Población, San José, Costa Rica; 17 Ministerio de Salud de la Provincia de Tierra del Fuego, Ushuaia, Argentina; 18 Instituto de Investigaciones Epidemiológicas Academia Nacional de Medicina, Buenos Aires, Argentina; 19 Ministerio de Salud y Ambiente, Buenos Aires, Argentina; 20 Department of Epidemiology and Public Health, University College London, United Kingdom; University of Colorado Denver, United States of America

## Abstract

**Background:**

Limited knowledge on the prevalence and distribution of risk factors impairs the planning and implementation of cardiovascular prevention programs in the Latin American and Caribbean (LAC) region.

**Methods and Findings:**

Prevalence of hypertension, diabetes mellitus, abnormal lipoprotein levels, obesity, and smoking were estimated from individual-level patient data pooled from population-based surveys (1998–2007, n = 31,009) from eight LAC countries and from a national survey of the United States (US) population (1999–2004) Age and gender specific prevalence were estimated and age-gender adjusted comparisons between both populations were conducted. Prevalence of diabetes mellitus, hypertension, and low high-density lipoprotein (HDL)-cholesterol in LAC were 5% (95% confidence interval [95% CI]: 3.4, 7.9), 20.2% (95% CI: 12.5, 31), and 53.3% (95% CI: 47, 63.4), respectively. Compared to LAC region’s average, the prevalence of each risk factor tended to be lower in Peru and higher in Chile. LAC women had higher prevalence of obesity and low HDL-cholesterol than men. Obesity, hypercholesterolemia, and hypertriglyceridemia were more prevalent in the US population than in LAC population (31 vs. 16.1%, 16.8 vs. 8.9%, and 36.2 vs. 26.5%, respectively). However, the prevalence of low HDL-cholesterol was higher in LAC than in the US (53.3 vs. 33.7%).

**Conclusions:**

Major cardiovascular risk factors are highly prevalent in LAC region, in particular low HDL-cholesterol. In addition, marked differences do exist in this prevalence profile between LAC and the US. The observed patterns of obesity-related risk factors and their current and future impact on the burden of cardiovascular diseases remain to be explained.

## Introduction

For the last two decades cardiovascular diseases have been the main cause of death in Latin America and the Caribbean (LAC). [1] Cardiovascular mortality rates continue to increase in most LAC countries, and in those countries where rates have declined the blunting of the trend has been considerably lower than in the United Sates (US). [2].

Data on the distribution of cardiovascular risk factors in LAC region are limited, and the few studies available show significant variation in the levels of prevalence. For instance, the CARMELA study, [3] conducted in seven major urban cities from LAC, reported markedly different hypertension levels. For instance, hypertension prevalence in Santiago (Chile), Buenos Aires (Argentina), and Barquisimeto (Venezuela), ranged from 24% to 29%, whereas in Quito (Ecuador), Bogotá (Colombia), Mexico City (Mexico), and Lima (Peru) varied from 9% to 13%. Yet, diabetes prevalence in these cities was similar to world’s estimates, around 7%. [3] Differences in rural-urban residence, socioeconomic development, and internal migration patterns could partly explain the contrasting profiles of cardiovascular risk factors, but knowledge on this regard is also very limited. This scarcity of data on the distribution of risk factors and, in turn, on their impact on incidence and mortality hampers efforts to curtail the growing epidemic of cardiovascular disease in LAC. In fact, national and regional health policies have been customarily based on estimates of the burden of risk factors and disease that rely heavily on demographic profiles. [4].

Here we report the distribution of cardiovascular risk factors using data from population-based studies from eight LAC countries. We also compare the distribution of cardiovascular risk factors in LAC and the US, as a way to illustrate the current stage of LAC in the process of the epidemiological transition. Insight into the specific differences in the distribution of risk factors in the LAC and US populations is important to foresee future trends in cardiovascular morbidity and mortality in the region.

## Methods

We analyzed data from the Latin American Consortium of Studies in Obesity (LASO), [5] and from the US National Health and Nutrition Examination Surveys (NHANES) 1999–2000, 2001–2002, and 2003–2004. [6] In total, LAC data from eight studies based on multi-stage random sampling and three studies based on single-stage random sampling were pooled. Only individuals ≥20 years old and non-pregnant women were included in our study.

Details on the LASO aims, methods and measurements have been previously published. [5], [7] Briefly, population’s size from the eight LAC countries included were as follow: Argentina (urban sample, n = 1076), Chile (national sample, n = 3461), Colombia (two studies, both urban samples, n = 4817), Costa Rica (national sample, n = 2826), Dominican Republic (national sample, n = 6117), Peru (three studies: national sample, n = 4201; urban/rural samples, n = 2868), Puerto Rico (urban sample, n = 865), and Venezuela (state sample, n = 4778). Details of each individual study, including year of study, location, sample, age ranges and sex distribution are available elsewhere [5]. Institutional Review Boards (IRB) in each country approved the individual studies and LASO was approved by IRB of the University of Wisconsin at Madison. All participants provided their informed consent. Standing height and weight were measured with the participants wearing light clothing and no shoes. Waist circumference (WC) was measured at the umbilical level in three studies, [8], [9], [10] at the midpoint between the lowest rib and the iliac crest in four studies, [11], [12], [13], [14] at the high point of the iliac crest in one study, [15] and was not measured in another. [16] Hip circumference was only measured in six studies, at the maximum extension of the buttocks. [8], [9], [10], [11], [14], [15] Blood pressure measurements were conducted at least twice in all but one study [12] following standard recommendations. [17] Blood samples were obtained after ≥8 hours of fast and serum glucose, total cholesterol, and high-density lipoprotein cholesterol were measured enzymatically by automated methods.

In NHANES, WC circumference was measured at the high point of the iliac crest and hip circumference was measured at the maximum extension of the buttocks. Three to four blood pressure measurements were taken following standard procedures [18], [19] and fasting blood glucose and lipids were also measured by automated methods. [20], [21].

Following standard practice, hypertension was defined as systolic blood pressure (SBP) ≥140 mm Hg, diastolic blood pressure (DBP) ≥90 mm Hg or self-reported current antihypertensive treatment. [22], [23] Diabetes mellitus was defined as fasting blood glucose ≥7.0 mmol/L (≥126 mg/dL) [24] or self-reported current pharmacological treatment for diabetes, i.e. insulin, oral hypoglycemic agents or both. High total cholesterol was defined as a serum total cholesterol level ≥6.15 mmol/L (≥240 mg/dL). High LDL-cholesterol was defined as ≥4.10 mmol/L (≥160 mg/dL), and low HDL-cholesterol as <1.03 mmol/L (<40 mg/dL) in men and <1.28 mmol/L (<50 mg/dL) in women. Hypertriglyceridemia was defined as a serum triglyceride level ≥1.69 mmol/L (≥150 mg/dL). [25] Current cigarette smokers were those who had smoked ≥100 cigarettes over their lifetime and smoked at the time of the interview in NHANES and two LASO studies, and those who smoke at least one cigarette per day at the time of interview in the remaining LASO studies. [11], [14] Overall obesity was defined as a body mass index (BMI) ≥30 kg/m^2^ and abdominal obesity as WC ≥88 cm in women and ≥102 cm in men. [26] In addition, we used alternative definitions for overall obesity (BMI ≥27 kg/m^2^) and abdominal obesity (WC ≥94 cm in women and WC ≥91 cm in men) based on cut-offs previously derived by our group for the LAC population. [7].

We used multivariate-chained equations to impute missing data and minimize selection bias due to the exclusion of participants with incomplete data in LASO (see Supplementary Materials S1). [27], [28] The imputation model was stratified by gender and included post-stratification weights, sociodemographic variables, anthropometric indicators, cardiovascular risk factors, and medical history of cardiovascular events. Ten complete datasets were generated and the parameters of interest were averaged across the imputed datasets using Rubin’s formula. [29].

We used post-stratification by age and gender distribution of the whole population of LAC countries included in the analysis as a way to lessen potential bias due to non-response and sampling frame under-coverage. [30] Post-stratification weights were constructed using the gender-by-age (10-year groups) distribution of the year 2000 population totals in each country. Individuals in the 70–79 and ≥80 years old strata were grouped together due to the reduced number of observations. Population size data were taken from the US Census Bureau (http://www.census.gov) for Puerto Rico and from the Centro Latinoamericano y Caribeño de Demografía - CELADE (http://www.cepal.org.ar/Celade) for all other countries.

We used multiple weighted linear and log-binomial regressions, clustering by country, to estimate the average level and the prevalence of each risk factor, as well as age and gender mean differences and prevalence ratios (PR). We used censored normal regression to estimate average blood pressure, as a way to correct for the bias that would have resulted from lower blood pressure levels among individuals receiving antihypertensive treatment (9% of the whole population). [31] We also explored potential age-dependent gender differences in the distribution of risk factors and adjusted for study to account for potential differences in study design and methods. Finally, we estimated age- and gender-adjusted PRs for each risk factor to compare each country to the whole LAC region.

The average level and the prevalence of cardiovascular risk factors in the US population were estimated in NHANES participants who attended to the mobile examination. We used the medical examination sampling weights to account for differential non-response and sampling frame under-coverage, and to adjust for over-sampling of some sociodemographic groups. [6] In order to compare the prevalence of risk factors in LAC and the US, we standardized the estimates from LASO and NHANES by the direct method to the age and gender distribution (20–29 to ≥70 years) of the civilian, non-institutionalized population of the US at the midpoint between 1999 and 2000. PRs were calculated from the standardized estimates and CIs were estimated using simulation. [32] Stata/MP 11 (Stata Corporation, College Station, Texas, US) was used for all analyses.

## Results

Our study included 31,009 individuals from LASO (mean age 39.9 years; age range 20–109; 95% CI: 38.2, 41.6; 49% men) and 13,441 individuals from NHANES (mean age 46.5 years; age range 20–85; 95% CI: 45.9, 47.1; 48.8% men).

### Distribution of Major Cardiovascular Risk Factors in LAC

#### Blood pressure

**Figure 1:**
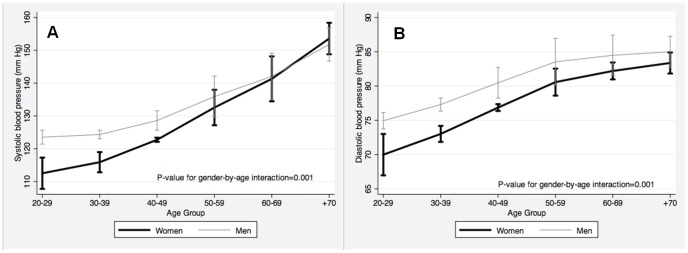
Estimated mean systolic and diastolic blood pressures by age and gender in the Latin American Consortium of Studies in Obesity (LASO). Panels show average blood pressure derived from censored normal regressions. All point estimates are provided with 95% confidence intervals. A) Systolic blood pressure (mm Hg); and, B) Diastolic blood pressure (mm Hg).

Mean SBP was 123.7 mm Hg (95% CI: 117.1, 130.3) in men and 119.0 mm Hg (95% CI: 113.6, 124.3) in women (p = 0.003). SBP increased with age in both men and women, but the rise was steeper among women (p-value for gender-by-age interaction: 0.003; Figure 1). As a result, before age 60, age-adjusted SBP was 5.6 mm Hg higher in men than in women (95% CI: 2.1, 9.0), whereas in participants ≥60 years men tended to have lower SBP than women (mean difference: −3.7 mm Hg; 95% CI: −7.6, 0.3).

Average DBP in men (76.9 mm Hg; 95% CI: 73.8, 80.0) was higher than in women (73.4; 95% CI: 70.5, 76.2; p = 0.005). DBP increased with age in both men and women, and was higher among men at all ages (Figure 1). However, the mean difference in DBP in men and women decreased with age, being 3.7 mm Hg (95% CI: 1.4, 6.0) in participants <60 years old and 0.2 (95% CI: −1.2, 1.7) in those ≥60 years old (*p*-value for gender-by-age interaction: 0.005).

**Figure 2:**
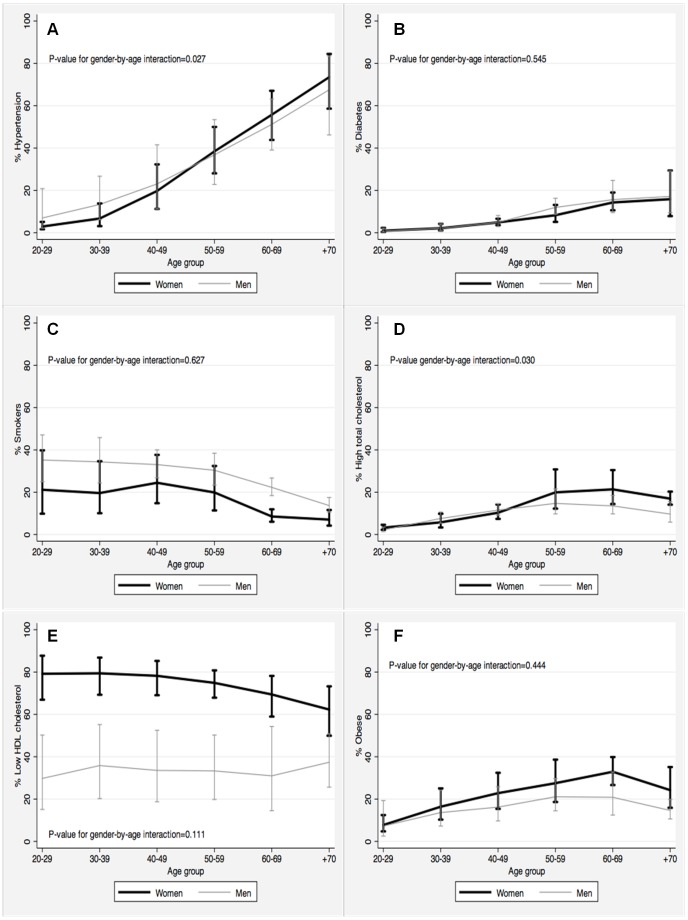
Prevalence of selected cardiovascular risk factors in the Latin American and the Caribbean population by age group and gender. All point estimates are provided with 95% confidence intervals. A) Hypertension; B) Diabetes; C) Smokers; D) High total cholesterol; E) Low HDL cholesterol; and, F) Obese.

The average prevalence of hypertension was 20.2% (95% CI: 12.5, 31.0), but varied considerably with age: from 5% in 20–29 years old to 70.9% in ≥70 years old (Figure 2). Overall, men were slightly more likely to be hypertensive than women (21.1% vs. 19.4%; Table 1). However, this gender disparity was considerable stronger among younger individuals. In fact, the PR was 1.68 (95% CI: 1.06, 2.65) in 20–29 years old but only 1.06 (95% CI: 0.98, 1.15) in those ≥70 years old (*p*-value for gender-by-age interaction = 0.027).

**Table 1:** Prevalence (%) of cardiovascular risk factor in the Latin American and the Caribbean population by gender.

Risk Factors	Women		Men		All	
	%	95% CI	%	95% CI	%	95% CI
**Hypertension**	19.4	(13.1; 27.6)	21.1	(11.9; 34.7)	20.2	(12.5; 31.0)
**Diabetes Mellitus**	4.8	(3.4; 6.8)	5.1	(3.5; 7.5)	5.0	(3.4; 7.9)
**High Total cholesterol**	9.6	(7.3; 12.7)	8.2	(6.4; 10.3)	8.9	(6.9; 11.4)
**High LDL cholesterol**	9.3	(6.0; 14.2)	7.6	(5.1; 11.0)	8.5	(5.8; 12.2)
**Low HDL cholesterol**	76.9	(68.2; 83.9)	32.8	(18.7; 51.0)‡	53.3	(47.0; 63.4)
**Hypertriglyceridemia**	23.3	(17.6; 30.2)	29.9	(20.1; 41.9)‡	26.5	(19.0; 35.7)
**Smoking**	19.5	(11.2; 31.9)	32.2	(25.5; 39.8)‡	25.8	(18.1; 35.3)
**Overall/Abdominal obesity**						
BMI ≥30 kg/m^2^	18.4	(13.5; 24.6)	13.8	(8.3; 21.9)	16.1	(11.1; 22.8)
BMI ≥27 kg/m^2^	33.1	(24.7; 42.7)	27.7	(18.4; 39.5)‡	30.5	(21.7; 40.8)
WC ≥88/≥102 cm (women/men)	55.5	(36.0; 73.4)	15.4	(7.2; 30.0)‡	35.8	(25.4; 47.8)
WC ≥94/≥91 cm (women/men)	22.8	(13.7; 35.4)	37.1	(21.7; 55.5)^§^	29.8	(17.9; 45.2)

‡ p<0.050 and § p<0.010 for prevalence ratios comparing men vs. women, adjusting for age and study. BMI: Body mass index; WC: Waist circumference.

#### Blood glucose

Mean fasting blood glucose was slightly but consistently higher in men (5.2 mmol/L; 95% CI: 4.8, 5.5) than in women (5.1 mmol/L; 95% CI: 4.8, 5.4; p = 0.052) across all age groups. The average prevalence of diabetes mellitus was 5% (95% CI: 3.4, 7.9; Table 1), but increased from 0.9% in 20–29 years old participants to 16.4% in those ≥70 years old. Across all age groups, men were slightly more likely to be diabetic than women, but not significantly so (age-adjusted PR: 1.14; 95% CI: 0.99, 1.32; Figure 2).

#### Serum lipoproteins

Mean total, HDL- and LDL-cholesterol were 4.73 (95% CI: 4.49, 4.96), 1.14 (95% CI: 1.09, 1.19), and 2.85 mmol/L (95% CI: 2.59, 3.11), respectively. Total cholesterol and both HDL- and LDL- fractions increased with age. After adjustment by age, total cholesterol was similar in men and women <50 years old. However, among participants ≥50 years, older women had significantly higher total cholesterol than men (mean difference: 0.25 mmol/L, p = 0.042). Likewise, LDL-cholesterol was higher in women than in men but only among individuals ≥70 years old (mean difference: 0.20 mmol/L, p = 0.020). In contrast, HDL-cholesterol was consistently higher in women than in men across all age groups (mean difference: 0.07 mmol/L, p = 0.016). On the other hand, average triglyceride levels were 1.70 mmol/L (95% CI: 1.53, 1.88) in men and 1.49 mmol/L (95% CI: 1.40, 1.58) in women (p = 0.028). This difference persisted after adjustment for age (mean difference: 0.22 mmol/L, p = 0.023).

The prevalence of high total cholesterol was about 9% (Table 1) and increased with age (Figure 2). High total cholesterol tended to be higher among young men and among older women than in corresponding groups of the opposite sex. PRs of elevated total cholesterol in men compared to women were 1.22 (95% CI: 0.97, 1.54), 1.12 (95% CI: 0.94, 1.33), 1.02 (95% CI: 0.89, 1.16), 0.93 (95% CI: 0.86, 1.06), 0.85 (95% CI: 0.72, 1.01) and 0.78 (95% CI: 0.62, 0.97) in participants 20–29, 30–39, 40–49, 50–59, 60–69, and ≥70 years old, respectively (*p*-value for gender-by-age interaction = 0.030). A similar pattern was observed for high LDL-cholesterol. Over one half of the population had low HDL-cholesterol (Table 1), but the prevalence was considerably and consistently lower in men (32.8%) than in women (76.9%), across all age groups (PR = 0.43; 95% CI: 0.24, 0.75; Figure 2). About a quarter of the population had hypertriglyceridemia (Table 1). Men had higher prevalence of hypertriglyceridemia, particularly at younger ages (*p*-value for gender-by-age interaction = 0.004). PRs for hypertriglyceridemia in men compared to women were 1.85 (95% CI: 1.36, 2.53) in participants 20–29 years old, 1.66 (95% CI: 1.28, 2.17) in 30–39 years old, 1.49 (95% CI: 1.20, 1.85) in 40–49 years old, 1.34 (95% CI: 1.12, 1.59) in 50–59 years old, 1.20 (95% CI: 1.05, 1.37) in 60–69 years old, and 1.08 (95% CI: 0.97, 1.20) in ≥70 years old.

#### Smoking

One out of four members of the LAC population were current smokers (Table 1). Smoking was considerably more prevalent in men than in women (32.2% vs. 19.5%, p = 0.006) regardless of age (p-value for gender-by-age interaction = 0.627; Figure 2). After adjustment for age, men were 1.59 times more likely to be current smokers than women (95% CI: 1.13, 2.23). Smoking prevalence decreased from 28.2% in 20–29 years old to 9.9% in ≥70 years old (Figure 2). Compared to 20–29 years old individuals, the gender-adjusted PRs for smoking were 0.95 (95% CI: 0.81, 1.11) in participants 30–39 years old, 0.96 (95% CI: 0.76, 1.22) in 40–49 years old, 0.82 (95% CI: 0.61, 1.11) in 50–59 years old, 0.49 (95% CI: 0.31, 0.79) in 60–69 years old, and 0.33 (95% CI: 0.17, 0.65) in ≥70 years old.

#### Overall and abdominal obesity

Mean BMI was 26.2 kg/m^2^ (95% CI: 25.1, 27.3) in men and 26.7 kg/m^2^ (95% CI: 25.9, 27.5) in women (p = 0.046). BMI increased with age, reaching maximum values at 50–59 years in both men (26.7 kg/m^2^) and women (28.0 kg/m^2^). BMI differences between women and men increased significantly with age from 0.6 kg/m^2^ (95% CI: 0.2, 1.1) in participants 50–59 years old to 1.4 kg/m^2^ (95% CI: 0.9, 1.9) in those ≥70 years old (p-value for gender-by-age interaction = 0.001). Correspondingly, the prevalence of overall obesity (BMI ≥30 kg/m^2^) was higher in women than in men: 18.4% vs. 13.8% (Table 1). Although the PR of obesity (women/men) seemed to increase with age (Figure 2) a gender-by-age interaction was not statistically significant (P = 0.444).

Mean WC was 90.8 cm (95% CI: 86.8, 94.8) in men and 87.2 cm (95% CI: 83.2, 91.2) in women (p = 0.015). After adjustment by age, the difference between men and women was 3.7 cm (95% CI: 1.2, 6.2), with no significant gender-by-age interaction (P = 0.273). The prevalence of abdominal obesity as defined by the World Health Organization (WC ≥88 cm in women and ≥102 cm in men) was more than three times higher in women than in men (55.5% vs. 15.4%) and the PR did not change significantly by age (age-adjusted PR = 3.6; 95% CI: 1.54, 8.35; *p*-value for gender-by-age interaction: 0.158). Gender-adjusted PRs for abdominal obesity were: 1.31 (95% CI: 1.02, 1.69), 1.58 (95% CI: 1.12, 2.22), 1.81 (95% CI: 1.27, 2.59), 1.94 (95% CI: 1.45, 2.61), and 1.77 (95% CI: 1.31, 2.39) for participants 30–39, 40–49, 50–59, 60–69, and ≥70 years old, compared to those 20–29 years old. When the definition of abdominal obesity was based on the ethnic and gender specific cut points derived from LASO (WC ≥94 cm in women and WC ≥91 cm in men) [7] the prevalence was higher in men than in women (37.1% vs. 22.8%) and the PR did not change significantly by age (age-adjusted PR = 1.67; 95% CI: 1.35, 2.06).

### Comparison between Each Country and the LAC Region

When compared to all the countries included in this analysis, Argentina showed similar prevalence of diabetes, high cholesterol, and smoking, but significantly lower prevalence of hypertension (PR = 0.61; Table 2). Chile had significantly higher prevalence of hypertension (PR = 1.48) and smoking (PR = 1.82). Colombia and Costa Rica, had significantly higher prevalence of high total cholesterol (PRs: 1.67 and 1.86), but significantly lower prevalence of smoking (PRs: 0.65 and 0.76, respectively). In turn, Peru had significantly lower prevalence of hypertension (PR: 0.65) and diabetes (PR: 0.41), while Puerto Rico had a significantly higher prevalence of diabetes (PR: 2.89). In general, the prevalence of each risk factor tended to be lower in Peru and higher in Chile compared to the region.

**Table 2:** Age- and gender-adjusted prevalence ratios for major cardiovascular risk comparing each country against all the countries from Latin American and the Caribbean included in this study.

Country	Hypertension	Diabetes	High total cholesterol	Smoking
Argentina	**0.61** *	1.12	0.84	0.41
Chile	**1.48** *	1.10	1.02	**1.82** *
Colombia	0.70	0.90	**1.67** *	**0.65** *
Costa Rica	1.15	1.40	**1.86** *	**0.76** *
Dominican Republic	1.31	1.25	0.83	0.74
Peru	**0.65** *	**0.41** *	0.75	0.68
Puerto Rico	1.19	**2.89** *	1.10	0.99
Venezuela	1.49	0.98	0.78	0.87

* 95% confidence interval does not include 1.00.

### Comparison between the LAC and the US Population

**Figure 3:**
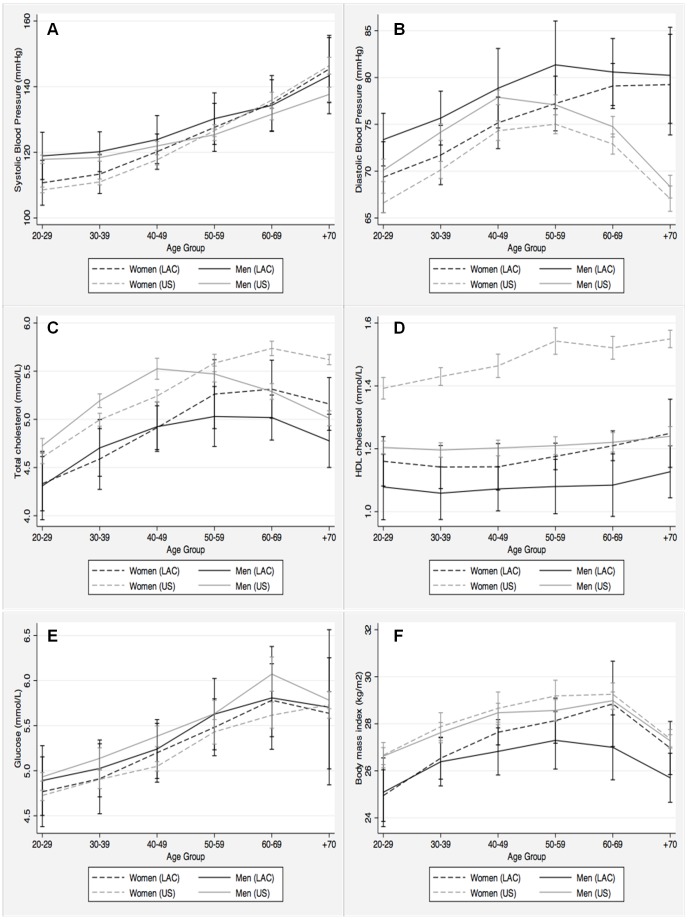
Mean values of selected cardiovascular risk factors in the Latin American and the Caribbean and the US population by age group and gender. All point estimates are provided with 95% confidence intervals. A) Systolic blood pressure (mm Hg); B) Diastolic blood pressure (mm Hg); C) Total cholesterol (mmol/L); D) HDL cholesterol (mmol/L); E) Glucose (mmol/L); and, F) Body mass index (Kg/m^2^).

Age-specific levels of SBP were slightly, but consistently higher in LAC than in US men and women (Figure 3). Interestingly, while in the US population SBP became higher in women than in men around age 50, in the LAC population this cross-over happened around age 60. This seems to be the result of a steeper age-related increase in SBP among US as compared to LAC women. DBP was also higher in LAC men and women for all age groups. However, DBP declined significantly starting at age 40 in US men and women, while in LAC men DBP flattened after age 50 and continued to increase among LAC women. Nevertheless, age and gender adjusted prevalence of hypertension was similar in the US and the LAC populations (Table 3).

**Table 3:** Crude prevalence (%) of cardiovascular risk factors in the United States and the Latin American and the Caribbean populations, and age- and gender-adjusted prevalence ratios.

Risk Factors	Unites States*		Latin Americans		PR†
	%	95% CI	%	95% CI	%
**Hypertension**	30.5	(28.8; 32.2)	20.2	(12.5; 31.0)	0.93
**Diabetes Mellitus**	8.2	(7.5; 8.8)	5.0	(3.4; 7.9)	0.86
**High Total cholesterol**	16.8	(15.8; 17.7)	8.9	(6.9; 11.4)	**0.64** §
**High LDL cholesterol**	13.8	(12.6; 14.9)	8.5	(5.8; 12.2)	**0.72** §
**Low HDL cholesterol**	33.7	(32.2; 35.2)	53.3	(47.0; 63.4)	**1.63** §
**Hypertriglyceridemia**	36.2	(34.4; 38.1)	26.5	(19.0; 35.7)	**0.82** §
**Smoking**	21.0	(19.7; 22.4)	25.8	(18.1; 35.3)	1.13
**Overall/Abdominal obesity**					
BMI ≥30 kg/m^2^	31.0	(29.5; 32.5)	16.1	(11.1; 22.8)	**0.58** §
WC ≥88/≥102 cm (women/men)	50.0	(48.2; 51.7)	35.8	(25.4; 47.8)	**0.79** §

* Data from the continuous NHANES (1999–2004).

† Age- and gender-adjusted prevalence ratios with US as reference.

§ 95% confidence interval does not include 1.00. BMI: Body mass index; WC: Waist circumference.

Age and gender-specific levels of total cholesterol were consistently higher in the US population than in the LAC population (Figure 3). Also, the gender-specific patterns of age-related changes in total cholesterol were similar in both populations. Accordingly, the age and gender-adjusted prevalence of high total cholesterol was about 25% lower in the LAC population (Table 3).

Regardless of age, HDL cholesterol was higher in men and women from the US than in men and women from LAC (Figure 3). HDL cholesterol level was higher in women than in men in both the US and the LAC populations, but the magnitude of this gender differences was considerably larger in the US. This was consistent with an age and gender-adjusted 63% higher prevalence of low HDL cholesterol in the LAC population (Table 3).

In contrast, age-specific levels of blood glucose were higher in US men, but lower in US women than in the corresponding LAC groups. Also, the age and gender-adjusted prevalence of diabetes was lower, but not significantly so, in the LAC population. Moreover, US men and women had higher BMI than LAC men and women, across all age groups (Figure 3). Accordingly, the age and gender-adjusted prevalence of overall obesity and abdominal obesity were significantly lower in the LAC population (PR: 0.58 and 0.79, respectively). Finally, the prevalence of smoking was similar in both populations (Table 3).

## Discussion

Our results indicate that the prevalence of major cardiovascular risk factors in the LAC region is within the range observed in developed countries, varying from 5% for diabetes to 30% for overall obesity. [33] We also found that after accounting for differences in the age distribution, the prevalence of hypertension, diabetes, and smoking were similar in the LAC and the US populations. [33] In contrast, the prevalence of obesity and high total cholesterol were significantly higher in the US, but the prevalence of low HDL cholesterol was significantly higher in the LAC population. We also found considerable between-country heterogeneity within the LAC region. The most striking departures from regional averages were a higher prevalence of hypertension and smoking in Chile, a lower prevalence of all risk factors in Peru, and a very high prevalence of diabetes in Puerto Rico, as compared to the whole group of countries included in this analysis.

In general, the prevalence of cardiovascular risk factors in the LAC population was within the range of prevalence observed in the US. Specifically, gender and age-adjusted prevalence of hypertension, diabetes, hypertriglyceridemia and smoking were only 10–20% lower in LAC, while the prevalence of total and high LDL-cholesterol, and abdominal obesity were 20–40% lower. The most striking differences between these two populations were a 42% lower prevalence of obesity and a 63% higher prevalence of low HDL-cholesterol in Latin Americans. An additional important finding was that despite very similar levels of SBP, LAC men and women had higher DBP levels than their US counterparts. Such finding was most evident among participants ≥50 years old. A marked age-related decline in DBP was observed in this age group in participants from the US but not in those from LAC; indeed, women from LAC demonstrated a continued increase in DBP after age 50. These findings generalize previous observations from a Peruvian population [34] to LAC and hint to important differences in the underlying hemodynamic determinants of blood pressure between the US and LAC populations. However, due to the cross-sectional nature of the data, it cannot be excluded that survival bias was at least partially responsible for these differences. The cardiovascular implications of a different pattern of age-related changes in blood pressure in LAC need to be addressed in longitudinal studies.

The prevalence of hypertension found in our study (20.2%) was similar to that reported by CARMELA (18%), [3] even though our study included individuals from non-urban settings. In contrast, our estimate of the prevalence of hypertension was only half that from a worldwide study on the burden of hypertension, that included reports from 1980 to 2002 (44% in men and 40% in women). [35] The prevalence of diabetes mellitus estimated in our study (5%) was within the range of that found in CARMELA [3], as well as that reported by the International Diabetes Federation. [36].

LAC women, particularly those in older age groups, were found to have a worst profile than men for several risk factors. This was most notorious for obesity-related traits such as overall and abdominal obesity indicators as well as low HDL-cholesterol. Furthermore, approximately one out of three post-menopausal women was obese, and prevalence of hypertension and low-HDL in older women were around 60%.

In age-adjusted analysis, despite the fact that overall and abdominal obesity were substantially less frequent in LAC than in the US, the prevalence of obesity-related traits such as hypertension and diabetes mellitus was similar in both populations, whereas the prevalence of low HDL-cholesterol was much higher in LAC (53.3% vs. 33.7%). This finding is unlikely explained by differences in the use of statins, as these drugs have only a minor effect on HDL-cholesterol. These observations suggest that metabolic disturbances associated with atherosclerosis arise at lower levels of adiposity in the LAC as opposed to the US population. Further investigation is required to ascertain whether genetic, environmental, or behavioural factors account for these differences, particularly those in HDL-cholesterol levels.

The prevalence of smoking in LAC population was 25.8% (19.5% in women and 32.2% in men). In both men and women, higher prevalence were observed in younger age groups, with a decline seen after age 50. The LAC pattern of smoking clearly contrasts with that observed in other low- and middle-income countries such as India, where higher prevalence of smoking is seen with increasing age. [37] This finding may reflect a birth-cohort effect, and has important implications, as it points to the need to develop and maintain anti-smoking campaigns that particularly target younger age groups in the LAC region.

Recent international reports have noted the limited availability of good quality surveillance data in most low- and middle-income settings. [33], [38], [39] Given that some surveys take longer, or do not make it to scientific reports, we had the advantage of collaborating closely with our colleagues from LAC region to expand the availability of data and provide more precise estimates for the LAC region. Indeed, about half (4/9) contributing studies to LASO published their results as local reports or publications in Spanish. We acknowledge that our findings may not apply to the whole LAC region, since several LAC countries, with large populations or with very unique set of prevention policies such as Brazil, Mexico and Uruguay, were not included in our study. Nevertheless, ours is the most comprehensive and representative regional study to date.

A particular strength of our study is the pooling of individual data from thousands of subjects, obtained from similar studies based on random samples of the population. Although the LASO studies did not share the exact same protocol, methods to measure anthropometric indicators, blood pressure, and blood glucose and lipids were similar, and should be a minor source of variability, which was partly accounted for by adjusting for study in all analyses. [5] By using multiple imputation we likely decreased the potential for selection bias and the loss of power that would have resulted from excluding from the analysis those individuals with missing values in one or more study variables. [28] Moreover, by post-stratifying by age and gender we were able to obtain prevalence estimates that are more representative of the whole population of LAC. Although single country estimates are desirable, they would have been unstable. Therefore, we gave preference to obtaining accurate regional estimates that should be useful in characterizing global health status and defining regional cardiovascular health policies. This is one of the few studies that included rural population and, therefore, provides a better picture of the cardiovascular risk profile in the LAC population. [40].

Although more detailed information is needed, particularly at the country level, our results provide further insights into the specific differences in the distribution of risk factors in the LAC and US populations. They should be useful for monitoring regional trends, as well as for defining and implementing more effective preventive strategies in the whole Americas region.

## Supporting Information

Materials S1 Description of the multiple imputation procedure in data from the Latin American Consortium of Studies in Obesity (LASO).
(PDF)
