# Supplementary material for: Major Cardiovascular Risk Factors in Latin America: A Comparison with the United States. The Latin American Consortium of Studies in Obesity (LASO)
Source: PLoS One. 2013 Jan 17;8(1):e54056. doi: 10.1371/journal.pone.0054056 (PMC3547948; doi:10.1371/journal.pone.0054056)
Supplement: Materials S1 — Description of the multiple imputation procedure in data from the Latin American Consortium of Studies in Obesity (LASO). (PDF) [file pone.0054056.s001.pdf]

SUPPLEMENTAL MATERIAL: METHODS

Description of the multiple imputation procedure in data from the Latin American Consortium of Studies in Obesity (LASO)[1]

I. Missing values for variable of interest

Table. Distribution of missing values in variables included in the multiple imputation model.

Variable	Missing		Random sample*		Not included in design@		Missed at exam†	
	No.	%	No.	%	No.	%	No.	%
Hip circumference (cm)	16692	53.8	3461	20.7	11394	68.3	1837	11.0
Serum glucose (mg/dL)	11064	35.7	704	6.4	8348	75.4	2012	18.2
Waist circumference (cm)	8767	28.3	704	8.0	6117	70.0	1946	22.0
Triglycerides (mg/dL)	7028	22.7	1685	24.0	1299	18.5	2984	42.4
Diabetic?	5569	18.0	687	12.3	0	0.0	4882	87.7
Weight (kg)	5341	17.2	704	13.2	0	0.0	4637	86.7
Height (m)	5345	17.2	704	13.2	0	0.0	4641	86.8
HDL-chol (mg/dL)	5284	17.0	1685	31.9	1076	20.4	2523	47.7
Total chol (mg/dL)	4821	15.6	2389	49.6	0	0.0	2432	50.4
Diabetes treatment	4239	13.7	684	16.1	0	0.0	3555	83.9
Systolic BP	2482	8.0	704	28.4	0	0.0	1778	71.6
Diastolic BP	2503	8.1	704	28.1	0	0.0	1799	71.9
Diabetes diagnosis	654	2.1	0	0.0	0	0.0	654	100.0
Hypertensive?	2337	7.5	662	28.3	0	0.0	1675	71.7
Hypertension diagnosis	569	1.8	0	0.0	0	0.0	569	100.0
Hypertension treatment	579	1.9	0	0.0	0	0.0	579	100.0
Current smoker?	183	0.9	0	0.0	0	0.0	183	100.0
Education level	171	0.6	0	0.0	0	0.0	171	100.0

*Some studies conducted the physical exam and/or lab test only in a random sample of all participants (Chile and Argentina); @ The original study design did not contemplate the measurement of this variable; † Subject did not show up for the physical exam or a blood sample was not provided.

II. Model details

Missing values were completed using multiple imputation by chained equations (MICE).[2] MICE is a fully conditional specification (FCS) method. FCS is a semi-parametric and flexible approach that specifies the multivariate model by a series of conditional models, one for each incomplete variable. FCS provides tremendous flexibility and is easy to apply, but its statistical properties are difficult to establish. However, simulation studies show that FCS is a good alternative when realistic joint distribution can be specified. The missing observations are assumed to be missing at random (MAR) or missing completely at random (MCAR). The imputation model was fitted using the command "ice" version 1.7.3, 07sep2009 from Stata 11.0 (Stata Corp).[3,4]

II.1 . Variables included in imputation model:
Study identification; age in years; sex; education level; weight (kg); height (m); waist circumference (cm); hip circumference (cm); systolic blood pressure; diastolic blood pressure; hypertensive status (current high blood pressure or antihypertensive treatment); previous diagnosis of hypertension; current pharmacologic treatment for hypertension; serum glucose (mg/dl); diabetic status (current high serum glucose or pharmacologic treatment); previous diagnosis of diabetes mellitus (not pregnant); current pharmacologic treatment for diabetes mellitus; total cholesterol (mg/dL); HDL-cholesterol (mg/dL); triglycerides (mg/dL); current smoking; fasting >=12 hours; hHistory of cardiovascular disease.

II.2. Log-transformed variables: age; height; systolic BP; diastolic BP; blood glucose; total cholesterol; ldl-cholesterol, and triglycerides.

II.3. Passively imputed variables: Hypertension was imputed passively and took the value 1=YES if the original hypertension variable was equal 1 or the log_sbp>=4.9416424 or the log_dbp>=4.4998097. Diabetes mellitus was imputed passively and took the value 1=YES if the original diabetes variable was equal 1 or log_glucose>=4.8362819.

II.4. Conditionally imputed variables: Hypertension treatment was imputed within the group of individuals with hypertension who had been diagnosed as such. Diabetes treatment was imputed within the group of individuals with hypertension who had been diagnosed as such. Conditional imputation occurs after imputation of conditioning variables.

II.5. Regression models:

- Linear regression was used for variables log_age, weight, log_height, waist circumference, log_glucose, hip circumference, log-systolic BP, log diastolic BP, log-glucose, log-Total cholesterol, log-hdl-cholesterol and log-triglycerides.
- Smoking, hypertension diagnosis, hypertension treatment, diabetes diagnosis, and diabetes treatment were modeled with logistic regression.
- Educational level was modeled as ordered logit.

Regression models included all variables. However, fasting state was excluded from the model for smoking status, hypertension treatment was excluded from the model for hypertension diagnosis, and diabetes treatment from the model for diabetes diagnosis. Body weight was modeled as an interval censoring variable, so that imputed weights cannot be smaller nor larger than the smallest and largest observed weight, respectively.{Royston P. Multiple imputation of missing values: Further update of ice, with an
emphasis on interval censoring. *Stata Journal* 2007, 7:445–464} Separate models were fitted for men and women, to allow for the inclusion of interactions with gender in future analyses.

II.6. Number of imputed data-sets:

A 100 datasets were imputed (the number used in a particular analysis could change)

II.7. Number of iteration cycles:

We used 200 cycles to attain convergence of the model. The minimum number of iteration cycles was determined visually from graphs of the mean the imputed variable against cycle number. A model with 350 cycles and 1 imputation was used for this purpose. Convergence was determined to occur at the cycle where the imputed mean was stabilized (didn't change further). Two examples of these graphs are provided below.

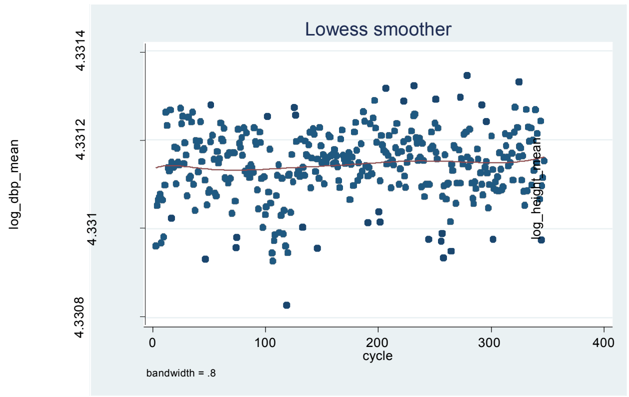

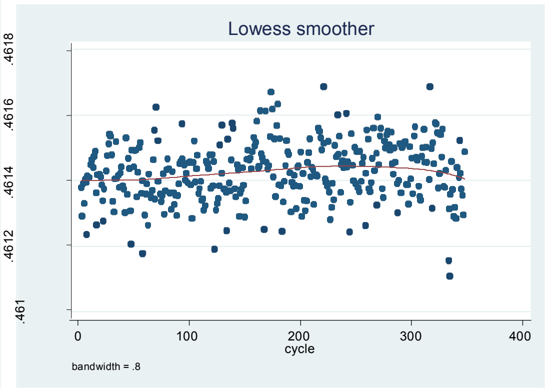

III. A comparison between imputed and non-imputed results

Table. Mean values for imputed and non-imputed variables (10 imputed datasets)

**Variable**	**Imputed**	**FMI**	**Complete data (non-imputed)**
Hip circumference (cm)	99.2 (95.5, 102.9)	0.054	100.1 (91.9, 108.2)
Serum glucose (mg/dL)	91.3 (83.1, 99.6)	0.000	91.3 (83.7, 99.0)
Waist circumference (cm)	88.9 (85.2, 92.7)	0.001	88.8 (83.8, 93.8)
Triglycerides (mg/dL)	149.5 (131.3, 167.7)	0.000	149.5 (132.5, 166.5)
Diabetic (%)	4.8 (2.9, 6.7)	0.001	5.95 (2.61, 9.30)
Weight (kg)	68.2 (64.9, 71.5)	0.000	68.6 (64.2, 73.0)
Height (m)	1.61 (1.59, 1,63)	.	1.61 (1.59, 1.63)
HDL-cholesterol (mg/dL)	44.0 (42.0, 46.1)	0.035	43.3 (41.0, 45.6)
Total cholesterol (mg/dL)	183.9 (169.8, 198.0)	0.000	183.9 (170.5, 197.2)
Diabetes treatment (%)	3.50 (0.61, 6.39)	0.003	2.99 (0.32, 5.65)
Systolic BP	120.4 (112.8, 128.0)	0.000	120.4 (113.2, 127.7)
Diastolic BP	74.7 (71.2, 78.2)	.	74.7 (71.4, 78.0)
Diabetes diagnosis (%)	4.36 (1.41, 7.30)	0.000	4.32 (1.56, 7.09)
Hypertensive? (%)	20.2 (10.5, 29.9)	0.008	25.4 (15.9, 35.9)
Hypertension diagnosis (%)	16.0 (6.8, 25.1)	0.000	15.9 (7.2, 24.6)
Hypertension treatment (%)	9.0 (5.0, 13.1)	0.000	9.0 (5.2, 12.8)
Current smoker? (%)	25.8 (16.6, 34.9)	0.000	26.0 (16.9, 35.0)
Educational level			
None	4.6 (0.0, 10.5)	0.000	4.6 (0.0, 10.3)
Primary school	40.0 (25.8, 54.3)	0.000	40.3 (26.9, 53.6)
Secondary school	30.3 (19.0, 41.6)	0.000	30.6 (19.4, 41.8)
Some college +	24.3 (11.8, 36.8)	0.000	24.5 (12.5, 36.4)

Notice that the two variables with meaningful differences in mean (diabetes and hypertension) were imputed passively, but the mean of the variables used for the passive imputation (serum glucose and diastolic and systolic blood pressure) were virtually identical.

IV. References

1. Bautista LE, Casas JP, Herrera VM et al. The Latin American Consortium of Studies in Obesity (LASO). *Obes Rev.* 2009;10:364-370.
2. van Buuren S. Multiple imputation of discrete and continuous data by fully conditional specification. *Statistical Methods in Medical Research.* 2007;16:219-242.
3. Royston P. Further update of ice, with an emphasis on categorical variables. *Stata Journal.* 2009;9:466-477.
4. Royston P. Multiple imputation of missing values. *Stata Journal.* 2004;4:227-241.
